# Community-based knowledge translation: unexplored opportunities

**DOI:** 10.1186/1748-5908-6-59

**Published:** 2011-06-06

**Authors:** Anita Kothari, Rebecca Armstrong

**Affiliations:** 1 Faculty of Health Sciences, and Schulich School of Medicine and Dentistry, University of Western Ontario, Health Sciences Building 222, London, Ontario, Canada, N6A 5B9; 2 Jack Brockhoff Child Health and Wellbeing Program, McCaughey Centre, Melbourne School of Population Health, University of Melbourne, Level 5/207 Bouverie St, Carlton 3010, Victoria, Australia

## Abstract

**Background:**

Knowledge translation is an interactive process of knowledge exchange between health researchers and knowledge users. Given that the health system is broad in scope, it is important to reflect on how definitions and applications of knowledge translation might differ by setting and focus. Community-based organizations and their practitioners share common characteristics related to their setting, the evidence used in this setting, and anticipated outcomes that are not, in our experience, satisfactorily reflected in current knowledge translation approaches, frameworks, or tools.

**Discussion:**

Community-based organizations face a distinctive set of challenges and concerns related to engaging in the knowledge translation process, suggesting a unique perspective on knowledge translation in these settings. Specifically, community-based organizations tend to value the process of working in collaboration with multi-sector stakeholders in order to achieve an outcome. A feature of such community-based collaborations is the way in which 'evidence' is conceptualized or defined by these partners, which may in turn influence the degree to which generalizable research evidence in particular is relevant and useful when balanced against more contextually-informed knowledge, such as tacit knowledge. Related to the issues of evidence and context is the desire for local information. For knowledge translation researchers, developing processes to assist community-based organizations to adapt research findings to local circumstances may be the most helpful way to advance decision making in this area. A final characteristic shared by community-based organizations is involvement in advocacy activities, a function that has been virtually ignored in traditional knowledge translation approaches.

**Summary:**

This commentary is intended to stimulate further discussion in the area of community-based knowledge translation. Knowledge translation, and exchange, between communities, community-based organizations, decision makers, and researchers is likely to be beneficial when ensuring that 'evidence' meets the needs of all end users and that decisions are based on both relevant research and community requirements. Further exploratory work is needed to identify alternative methods for evaluating these strategies when applied within community-based settings.

## Background

Knowledge translation (KT) is an interactive process of knowledge exchange between health researchers and users [1]. The area of KT has received much attention from researchers, governments at various levels, and research funding bodies of late. Ultimately, it is expected that the use of research in decision making will lead to a more efficient and effective health system, with longer-term positive impacts on the health of the population. Given that the health system is broad in scope, it is important to reflect on how definitions and applications of KT might differ by setting and focus. This commentary provides a critical reflection on KT as applied to community-based organizations. These, we argue, operate in unique circumstances that may impact on the processes by which KT might best be undertaken. Community-based KT is of interest to those community-based organizations involved in the delivery of health and health-related services with communities and populations often at the centre of intervention efforts. This includes, but is not limited to, public health departments, community health centres, and local health authorities. While perhaps not directly involved in the delivery of services, one might argue that non-governmental organizations, civil service organizations, and the voluntary sector also require special attention with respect to KT processes. Research in this area is just starting to emerge [2-8], and it is our intention to flag this work to stimulate further discussion in the area. Community-based organizations also play an important role in the delivery of health strategies that may have occurred as part of higher-level KT decision-making processes or policies. As such, they may provide important perspectives on the KT process. The objective of this article is, therefore, to differentiate and contextualize the term 'community-based KT' in order for KT processes in this domain to adequately capture the connection between evidence, decision makers, practitioners, and the communities they serve.

## Discussion

Until now, KT has primarily been studied from a medical decision-making perspective [9]. Most would agree that this perspective has evolved from the evidence-based medicine movement, defined as 'the conscientious, explicit, and judicious use of current best evidence in making decisions about the care of individual patients. The practice of evidence-based medicine involves integrating individual clinical experience with the best available external clinical evidence from systematic research' [10]. Decisions made in this context generally focus on the health outcomes of individual patients and usually assess changes in specific clinical behaviours (*e.g*., prescribing). The general issue is how to best facilitate individual level change within supportive environments [11]. Specific frameworks have been developed to understand how to change clinical behaviours [12], and tools generated to assist with decision making in clinical environments, such as clinical practice guidelines [13,14]. Using these frameworks and tools, a number of KT strategies have been implemented in this setting (see Table 1).

**Table 1:** Strategies implemented in clinical settings

Reminders and computerized decision support
Dissemination of educational material

Audit and feedback

Educational outreach

Opinion leaders

Computer systems

Feedback of cost data

Mass media

Source: Grimshaw JM, Shirran L, Thomas R, Mowatt G, Fraser C, Bero L, Grilli R, Harvey E, Oxman A, O'Brien MA: **Changing provider behavior: an overview of systematic reviews of interventions**. *Med Care 2001*, **39**(8 Suppl 2):II2-45.

The appropriateness and effectiveness of these strategies in other health settings is less well understood. For example, few of these have been evaluated rigorously in the public policy setting [1], and evidence that these strategies work in community-based organizations is just as limited [5]. In informing debates about the application of these strategies to alternative settings, we submit that community-based health settings are different from the clinical milieu, and this has implications for the study and application of KT approaches.

Differences in settings, in what is considered 'evidence,' and in outcomes of interest (see Table 2) suggest it might be worth reflecting critically on the appropriateness of the application of clinical KT strategies in community-based organizations [15]. Community-based organizations and their practitioners share common characteristics, described below, that are not satisfactorily reflected in current clinically focused KT approaches, frameworks, or tools [6].

**Table 2:** Differences in clinical and community-based settings

	Clinical	Community
Settings	Single practitioner or organization	Multi-organization involvement

		Clear value orientation

Evidence	Curative	Prevention and health promotion

	Clear focus on randomized controlled trials as best 'evidence'	Broad consideration of what is 'evidence'

Outcomes	Individual level interventions	Individual, community and population level interventions

	Individual level outcomes	Individual, community and population level outcomes within complex systems

		Advocacy outcomes

Source: Mitton C, Adair CE, McKenzie E, Patten SB, Perry BW: **Knowledge transfer and exchange: Review and synthesis of the literature**. *The Milbank Quarterly 2007*, **85**(4): 729-768. Green LW: From Research to 'Best Practices' in Other Settings and Populations [*]. *Am J Health Behav 2001*, **25**(3):165-178. (pg. 229). Wandersman A: Community Science: **Bridging the Gap between Science and Practice with Community Centered Models**. *Am J of Comm Pys 2003*, **31**(3-4):227-242.

### Settings: working collaboratively within and across organizations

Community-based organizations tend to value community strengths and the process of working in collaboration with stakeholders in order to achieve an outcome. This may include other organizations or the community more broadly. For example, community health centres and local authorities may work in collaboration with schools to deliver a healthy eating initiative. In some cases, this way of working is mandated in legislation. The Ontario Public Health Standards, for example, have outlined foundational principles that include working in 'extensive' partnership and collaboration with groups from multiple sectors [16]. As noted by Miller and Shinn, 'interrelationships among organizations may further constrain their autonomy to make decisions about their own activities' [17]. For example, there may be a strong history of service delivery patterns in particular settings, with specific population groups, or to address particular issues. De-investing in some approaches, regardless of their impact on health outcomes, may be difficult given this historical investment or 'relational capital' [6].

This approach to working has implications for traditional conceptions of KT related to research dissemination and subsequent application using strategies described in Table 1. How do such collaborations acquire, assess, adapt, and apply evidence? Strategies based on electronic reminder systems or audit and feedback are not viable options for influencing evidence-informed decision making in a non-hierarchical forum that values consensus building. Simply put, there is no 'gold standard' for how such collaborations ought to operate, making it difficult to imagine implementing and evaluating a standardized prompting system. Frameworks might be helpful to guide such work, but only a handful related to KT and community-based organizations were located in our search of the literature [5,18]. On the other hand, there may be room to assess the effectiveness of educational strategies or opinion leaders on partnership-based decision making. We recognize that these distinctions between clinical and community-based settings represent ideal types. The point is, however, that perhaps as community-based KT researchers we have limited our thinking about potential strategies because we have been tethered to the evidence-based medicine paradigm. Clearly this is an area in need of further systematic inquiry.

### Evidence: what research is available and what is considered evidence

Clinical settings tend to take on a curative approach to care, while community-based organizations lean towards prevention and health promotion activities (there are, of course, exceptions to this generalization). Green notes that curative-type interventions are likely to demonstrate similar outcomes across individuals, after adjustments for age, gender, and weight [19]. Thus efficacy study findings based on rigorous randomized controlled trials (RCTs), cohort studies, and case control studies are regarded as desirable evidence. By its very nature, however, similarly designed research on prevention and health promotion activities is not as readily available as might be for medical interventions [20]. This lack of research evidence makes it difficult for community-based organizations to create or seek out systematic reviews on their topic of interest, a precursor to implementing any of the KT strategies listed in Table 1. Alternatively, the gap between efficacy and effectiveness studies may be larger for population-level interventions than biomedical ones, leading to the erroneous conclusion that KT implementation failure occurred [21].

Related to this issue of research evidence is the desire for local information. Community-based organizations regularly engage in their own research -- needs assessments, capacity/asset mapping, focus groups, surveys -- with target populations. This type of research has been recently criticized for not being related to the broader literature base [5], or not mapping well onto 'evidence hierarchies' (RCTs, *et al*.). We wonder, in contrast, if the preference for local information stems from epistemological differences [2,22], concerns about generalizability related to RCTs, or the lack of expertise and resources required to access the formal literature. In our experience, the information from local research efforts is highly valued for its contextual relevance, and is perhaps more likely to be put into action through health programs. For KT researchers, developing processes to assist community-based organizations to adapt research to local circumstances may be the most helpful way to advance decision making in this area [23]. Further, increasing the rigor of local research may result in building a culture supportive of evidence-informed decision making.

These issues are further compounded by the multi-sectoral nature of the community partnerships and the way in which evidence is conceptualized by partner-stakeholders, which in turn influences the degree to which research evidence is relevant and useful [8,22]. While economic modeling, epidemiological evidence, or RCTs may represent the most influential forms of evidence in some sectors, in other sectors community views are a necessary component of collaborative partnerships and therefore represent sources of information. This information might include views about preferences (*e.g*., having the right to smoke in certain public places) or about experiences and insights related to health services, health states, or practices (*e.g*., breast self-examination makes women feel like they are in control despite research demonstrating its ineffectiveness). Whether this information is seen as important is value-laden, and it is likely that community-based organizations will consider such information as legitimate evidence to inform decision making. For example, a community-based organization may continue to encourage women in their communities to perform breast self-examinations because women have described the merit of the practice. Community-based organizations often face political and community pressure to address emerging needs, in which case tacit knowledge (field experience and professional expertise) may be preferred and highly valued [24]. Traditional KT frameworks, such as the Promoting Action on Research Implementation in Health Services (PARIHS) model [12], as well as the evidence-based decision making movement [10], do acknowledge patient preference as being a factor for the clinician to consider. However, we suggest that 'what is considered evidence' [22] by community-based organizations includes tacit knowledge, community views, and perhaps other sources of information yet unidentified. Emerging frameworks support this approach [25].

### Outcomes: networks and advocacy in community-based KT

KT strategies, such as reminder systems and audit and feedback, are feasible to evaluate because they focus on a definable behavior. In other words, assessing the outcomes desired from the strategies listed in Table 1 is facilitated by change in individual practice patterns, which in many cases can be obtained from documented sources such as medical charts. It is also feasible to measure community prevalence rates of treatments, such as caesarean sections, to determine if a promoted change in treatment has indeed been taken up by practitioners.

In contrast, KT strategies that promote certain prevention and health promotion activities are somewhat more difficult to assess [15,21]. As Green *et al*. state, '...the 'intervention' usually becomes increasingly a program made up of multiple interventions and the object is a diverse population or a community with heterogeneity across geographies, cultures, social structures, and histories' [21]. If traditional KT strategies are applied to community-based organizations, outcome measurement must consider networks of organizations and/or individuals who make collaborative decisions involving the use of a broad array of evidence for the collective delivery of services, not simply a solitary user of research. Measuring the change in 'practice' of a collaboration -- a KT outcome -- is difficult to carry out. Even if measuring the change in 'practice' of a collaboration is feasible, measuring change at the community level is difficult. Multiple factors contribute to prevention and health promotion outcomes, making it difficult to establish a link between a KT strategy and improved community health. Exploratory work is needed to identify alternative methods for evaluating these strategies when applied within community settings. This is likely to require multi-level data collection, with a strong emphasis on methodological pluralism [20], allowing community-based organizations to share their experiences with these processes [2]. In other words, KT strategies need to work collaboratively and sensitively with community-based organizations to build their capacity and promote an evidence-informed approach to decision making, where evidence is broadly defined.

Any alternative method for assessing community-based work needs to incorporate the key activity of advocacy for public policy on behalf of the populations served. This advocacy work, however, has thus far gone unnoticed in the traditional KT literature. Specifically, advocacy has not yet been framed as an outcome related to the utilization of knowledge. Traditional KT outcomes tend to be related to Weiss' conceptual, instrumental, or symbolic use of research [26], or a staged approach to the utilization of research [27]. Re-conceptualizing advocacy as a KT activity presents a tremendous opportunity to introduce sound evidence into the lobbying and ultimately the policy-making process.

In fact, we contend that advocacy represents an opportunity for meaningful exchange between communities, researchers, practitioners, and decision makers. This could involve partnerships where the community is involved in making decisions, but could also involve asking community-based organizations to represent the views of communities in higher-level decision-making processes [18]. In a sense, community-based organizations may act as brokers between researchers and communities. Previous research has identified the importance of quality relationships and trust in collaborative KT partnerships [28]. This may involve KT researchers working with community-based organizations to explore the opportunities and options for using evidence for advocacy purposes.

### Moving Forward

While the focus of this article has been on community-based organizations, there might be some clinical settings that share some of the characteristics described above. There may be practice settings that interact with stakeholders or networks, or that function in a non-hierarchical manner. Others might argue that a lack of relevant research is also a challenge for clinical practitioners; tacit knowledge may be extremely important, and welcome, in such situations. We also acknowledge that KT has occurred with some prevention and health promotion interventions. The underlying point of this article, however, remains the same: that there are health service delivery systems for which traditional ways of approaching KT are insufficient.

We note that the extensive community-based participatory research (CBPR) body of work provides an excellent starting point for working with community members and evidence. For example, this literature points to the importance of structures, processes, relationships, and principles emerging from CBPR studies that could inform future KT initiatives [18]. Yet, we know little about how to carry out effective KT when related to community collaborations with, within, and between health agencies. Further research should seek to identify and address partnership barriers and develop solutions that enable exchange.

### Summary

KT (and exchange) between communities, community-based organizations, decision makers, and researchers is likely to be beneficial when ensuring that 'evidence' meets the needs of all end users, and that decisions are based on both relevant research and community requirements. For community-based organizations, the challenge of combining a range of sources of evidence only increases the importance of exchange and collaboration among stakeholders. Meaningful exchange may also result in community-based organizations valuing and being able to resource rigorous evaluations and subsequently contributing to the larger literature base. While we advocate for the implementation of community-based KT and building the evidence about what works, we acknowledge the difficulties in measuring these outcomes. In the meantime, we welcome further discussion about the meaning and use of evidence in this setting, identification of the relevant actors, and ideas about potentially promising community-based KT strategies and outcomes.

## Competing interests

The authors declare that they have no competing interests.

## Authors' contributions

The concept of this manuscript was conceived by both AK and RA. Both authors contributed to the writing, editing and completion of the manuscript. Both authors have approved the final version of this manuscript.
